# Conscientious Objection to Legal Abortion in Minas Gerais State

**DOI:** 10.1055/s-0040-1721683

**Published:** 2020-11-30

**Authors:** Roger William Moraes Mendes, Antonio Marcos Coldibelli Francisco, Carla Benedita da Silva Tostes, Júlia dos Reis, Augusto Castelli Von Atzingen

**Affiliations:** 1 Faculdade de Medicina de Itajubá, Itajubá, MG, Brazil; 2 Department of Bioethics, Universidade do Vale do Sapucaí, Pouso Alegre, MG, Brazil; 3 Department of Obstetrics and Gynecology, Hospital de Clínicas de Itajubá, Itajubá, MG, Brazil

**Keywords:** bioethics, legal abortion, sexual violence, induced abortion, ambulatory care facilities, bioética, aborto legal, violência sexual, aborto induzido, instituições de assistência ambulatorial

## Abstract

**Objective** The aim of this study was to verify the existence of conscientious objection to comprehensive health care for the victim of sexual violence, as well as to understand the service structure of institutions authorized in the health care system for victims of sexual violence in the state of Minas Gerais.

**Methods** This is a quantitative, cross-sectional, descriptive, and analytical field study aiming to collect data from institutions authorized to assist victims of sexual violence in the state. The instrument was handed in to the coordinators of these services.

**Results** It was found that 11% have no physician in service and that 31% had no training for this type of care. It was revealed that 85% of these institutions have already encountered patients wishing to have a legal abortion, but 83% of them have not had their request granted. There was a 60% presence of conscientious objection by the entire medical team, the main reason being religious (57%).

**Conclusion** The assistance system is not prepared for comprehensive care for victims of sexual violence, especially in terms of legal abortions, with conscientious objection being the main obstacle. A functional referral and counter-referral system is needed to alleviate such a serious and evident problem. It is hoped that the research results will promote dialogues in the state that favor appropriate actions on legal abortion, and respect the medical professional, in case of conscientious objection.

## Resumo

**Objetivo** O objetivo do estudo foi verificar a existência da objeção de consciência na atenção integral da saúde à vítima de violência sexual, bem como conhecer a estrutura de atendimento das instituições credenciadas na rede de atenção à vítima de violência sexual no Estado de Minas Gerais.

**Métodos** Trata-se de um estudo de campo de caráter quantitativo, transversal, descritivo e analítico, com proposta de coleta de dados das instituições credenciadas ao atendimento às vítimas de violência sexual no estado. O instrumento foi entregue às(aos) coordenadora(es) destes serviços.

**Resultados** Verificou-se que 11% dos serviços não possuem médicos e 31% não fornecem treinamento para este tipo de atendimento. Foi revelado que 85% dessas instituições já encontraram pacientes que desejam fazer o aborto legal, mas 83% destas não tiveram seu pedido atendido. Houve 60% da presença de objeção de consciência por parte de toda a equipe médica, sendo o principal motivo religioso (57%).

**Conclusão** O sistema de assistência no Estado não está preparado para o atendimento integral às vítimas de violência sexual, principalmente no quesito resolução do aborto legal, sendo a objeção de consciência o maior obstáculo. Se faz necessária uma rede de referência e contra referência funcionante para amenizar problema tão sério e evidente. Espera-se que o resultado da pesquisa crie espaços de diálogos dentro do estado que favoreçam ações adequadas sobre o aborto legal, e o profissional médico respeitado, se houver objeção de consciência.

## Introduction

Abortion, a serious public health problem, is present in Brazil and is a subject that generates several discussions, both for the defense of its legalization and for the partial or unrestricted maintenance of its prohibition. 1 Despite the fact that the academic training of health professionals includes several approaches on this topic, the influences of ethical, moral, socioeconomic, political, cultural and religious issues bring difficulties to face it. As a topic discussed in the field of the so-called “bioethics of persistent situations,” abortion provides reflections about the autonomy of women over their bodies, the health professional's view on such decisions, and the ethical-political implications for the public health field. 2

According to the World Health Organization criterion adopted by Brazil, abortion is characterized by the termination of pregnancy until the 20th week, provided that the product of conception - the abortion itself - weighs less than 500 g. 3

In Brazil, the Penal Code 4 classifies rape as a crime against sexual freedom (article 213), to protect the victim's sexual dignity. In recent years, political, social, and judicial sectors have paid attention to victims of sexual violence. Such mobilizations resulted in the creation of assistance services for women victims of sexual and domestic violence, as well as legal instruments, such as Maria da Penha Law, 5 which allows more humanized care to these women.

In an attempt to guarantee the reproductive and sexual rights of women, defined in international agreements and signed in the national legislation in force, Brazil, through the Ministry of Health, published in 1999 the technical standard “Prevention and treatment of injuries resulting from sexual violence against women and adolescents” 6 to ensure the right to interrupt legal pregnancy through its Unified Health System.

It is known that conscientious objection is provided for in several normative acts or professional codes, embodied in the Federal Constitution, with the scope of protecting individuals in situations contrary to their moral principles. However, this right is not absolute when there is damage to other people's health. 7 Therefore, the right to conscientious objection finds limits, and it is not possible for professionals to invoke it in situations considered to be urgent, which are: risk of death to the pregnant woman; legally permitted abortion, in the absence of another professional who does it; possibility of the woman suffering damage or health problems due to the omission of the health professional, and complications resulting from unsafe abortion. 8

It is worth mentioning that Article 11 of the Universal Declaration on Bioethics and Human Rights 9 links the principle of non-discrimination and non-stigmatization to the principle of human dignity, which is why no one should suffer any embarrassment or be diminished by whatever they do or choose, under penalty of having their dignity removed. Although the alleged suffering of the woman who aborts and of the professional who performs it cannot be compared, it should be noted that all those involved in an abortion, even a legal abortion, seem to be vulnerable to different processes of stigmatization and discrimination. 8

Therefore, the aim of the study is to verify the existence and the reasons of conscientious objection to comprehensive health care for victims of sexual violence, with an undesired pregnancy and willing to interrupt it in Minas Gerais. Another aim of this study is to access the service structure of institutions authorized in the health care system for victims of sexual violence, verify the frequency of conscientious objection in these services and identify how the health care unit approaches this problem of legal abortion and comprehensive care for women victims of sexual violence.

## Methods

This is a quantitative, cross-sectional, descriptive and analytical field study. The investigation was performed in the years 2018 and 2019 from the survey of institutions accredited by SES / MG to assist victims of sexual violence in Minas Gerais. Then, all the coordinators of these 87 services were contacted to telephone calls, e-mails or telephone app (WhatsApp) and invited to participate in the research, previously approved by the ethics and research committee (opinion 3.584.672), however only 49 hospitals accepted the invitation. They then received the free and informed consent form together with the structured, specific and objective instrument, by email or link, according to the respondent's preference.

The questions were aimed at verifying whether the institutions have a reserved and exclusive environment for the care of victims of sexual violence, specific exams for this type of care, STD prevention, emergency contraception, training, humanization and welcoming care, and whether they have multiprofessional team. In addition to the structural aspect, it was asked about the occurrence of cases of victims of sexual violence, with unwanted pregnancies, wishing to terminate the pregnancy, as well as the presence or absence and reasons for the team's conscientious objection and what is the conduct in this situation. The data were allocated in a spreadsheet, using specific software (Microsoft Office - Excel ®), performed Chi-Square test, Yates test and direct percentage.

## Results

Note that 49 out of the total of 87 authorized institutions responded to the request to participate in the study. Of the 49 institutions that answered the questionnaire, 14 were excluded from the analysis because they do not offer maternity services at the facility and or do not know whether they are authorized by SES/MG for comprehensive care for victims of sexual violence. Thus, 35 hospitals were evaluated in the present study.

The 38 institutions that did not respond to requests by telephone calls, e-mails, or smartphone application (WhatsApp) were not included, as these did not respect the inclusion criteria of the research.

Table 1 presents the results referring to the first question of the instrument, regarding the availability of services and the structure of maternity hospitals accredited for the care of victims of sexual violence, by SES / MG.

**Table 1:** Available services and structures of Minas Gerais

Services and structures	Yes(%)	No(%)
Psychologist	28(80,00)	7(20,00)
Physichian	31(88,58)	4(11,42)
Nurse	29(82,86)	6(17,14)
Social worker	30(85,71)	5(14,29)
Private área	11 (31,43)	24 (68,57)
Tests	30(85,71)	5(14,29)
Training	24(68,57)	11(31,43)

**Fig. 1:**
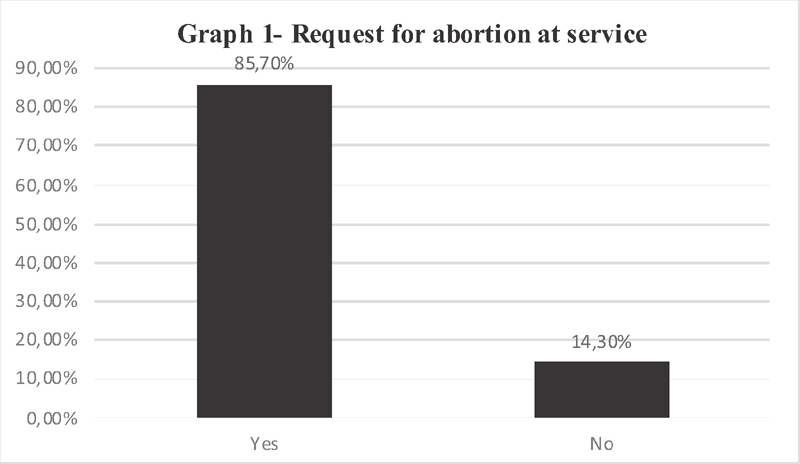
Situations of assistance to the victim of sexual violence, with unwanted pregnancy or anencephaly, with the desire to terminate the pregnancy.

When asked about assistance to victims of sexual violence, with unwanted pregnancy or anencephaly, wishing to terminate the pregnancy, 30 (85,70%) responded that they had already met this circumstance, while 5 (14.30%) answered no, as shown in Fig. 1 .

**Fig. 2:**
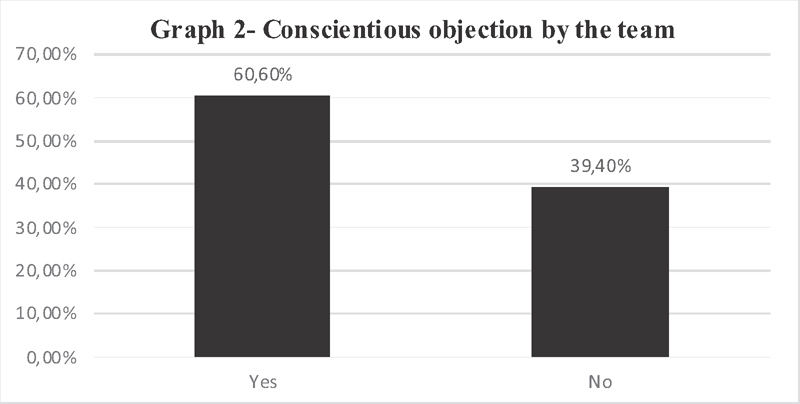
Conscientious objection by the whole team to carry out legal abortion according to maternity coordinators authorized by SES/MG.

Regarding conscientious objection ( Fig. 2 ) in the performance of legal abortion by the entire team, 21 (60,60%) of the coordinators of the maternity hospitals authorized to care for victims of sexual violence by SES/MG replied that there is conscientious objection in this situation, and 14 (39.40%) answered no.

**Fig. 3:**
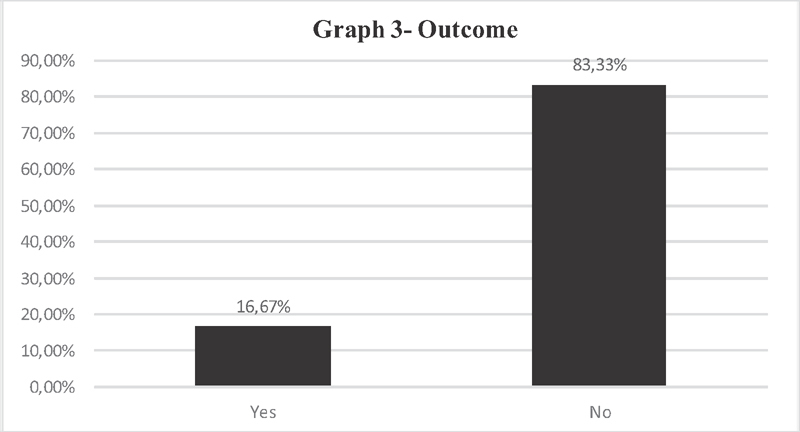
Outcome of positive demands for the desire for legal abortion in victims of sexual violence in maternity hospitals authorized by SES/MG.

Fig. 3 shows the percentage referring to the resolution or not, only of those who claimed to have already had a demand for a legal abortion in care for victims of sexual violence. The performance of the abortion is included in 5 services (16,67%) who answered “yes,” while 25 hospitals (83,33%) did not solve the problem. These services referred the problem to be solved by the hospital administration, or to a referred hospital, or to guide the patient herself to look for another service.

Table 2 shows the reasons mentioned for the conscientious objection by on duty at the maternity hospitals authorized to assist victims of sexual violence in SES/MG. Only those who presented conscientious objection by the whole team (21 services) answered this question. Note that in this question, those responsible for the services could mark more than one reason for objection.

**Table 2:** Reason for conscientious objection

Reason for objection	Yes (%)	No (%)
Religious	12(57,14)	9(42,86)
Ethical	11(52,38)	10(47,62)
Moral	7(33,33)	14(66,67)
Social	6(28,57)	15(71,43)
Always against abortion	8(38,10)	13(61,90)

## Discussion

The data collected at SES/MG showed that there are 87 services authorized to assist victims of sexual violence, and 14 of them were excluded from the research, not statistically entering the result and not answering the data collection instrument, as there was no maternity unit nor this type of service; that is, 16,09% of the total number of authorized institutions were excluded from the analysis. This relevant data must be debated, since 16,09% of authorized hospitals or municipalities do not provide the service they have been licensed to. 10

Table 1 show the deficiency and disruption of such essential care for women victims of sexual violence in Minas Gerais. The results reveal that the services do not fulfill the basics, which would be a private, separate, confidential, and humanized room, exclusive for this kind of service. These women arrive at maternity hospitals that receive patients in labor, with their families and caregivers, most of the time with crowded receptions and with no preparation to assist these frail and victimized women. 11 Still within these results, part of the services showed they do not have physicians (11,42%) and nurses (17,14%), which would be the minimum expected in a care unit for victims of sexual violence. 12

Analyzing the services provided, 14,29% of them do not have specific tests, STD prevention and emergency contraception, which are also basic and essential conditions to care for the victims of sexual violence according to the Resolution SES/MG No. 4,590, of December 09, 2014. 13 In addition, services need to be constantly updated, with training, humanization, and care for these women, with 31,43% of them denying this type of continuing education.

In the facilities surveyed and authorized to care for victims of sexual violence in Minas Gerais, 85.70% of them ( Fig. 1 ) have already encountered situations of care, with women who sought the service to legally terminate pregnancy. This problem is common, and the right of women is violated, as the law stands, in cases of rape, anencephaly and risk of maternal death, in all three situations, with no need for court approval. 14

It was identified that 60.60% of the researched institutions had conscientious objection by the whole team ( Fig. 2 ), that is, in these services the patient's problem is not, and probably will not be solved. This is of greatest importance at work, as it shows that more than half of the authorized facilities do not perform the medical act of legal abortion, because there is conscientious objection to this procedure by the whole team. This fact agrees with published studies reporting that there is a reduced number of professionals available to perform the procedure in Brazil. 15 Therefore, it is clear the conflict between physicians who have the right not to perform the act of abortion and women who have the right to legal abortion. Identifying this index at national level is essential to create focal points for political discussion on real comprehensive assistance to women. The statistic indicates that 35 services do not solve a problem that must be protected by law if it is taken into consideration 21 services that present conscientious objection by the full team ( Fig. 2 ) and 14 of the hospitals that were excluded from the research. This data reveals that 71.42% of the 49 hospitals that responded to the survey do not fully care for the victim of sexual violence.

Analyzing the outcome of the request of women who opted for legal abortion in authorized institutions in Minas Gerais, respecting the law and the desire to solve their problem, 16.67% of the coordinators in authorized maternity hospitals responded that when faced with women opting for legal abortion, they performed the medical act of abortion, but on the other hand, 83,33% of them did not meet the patients' wish; that is, 83,33% of these women could not have a legal abortion, which should be covered by law ( Fig. 3 ). In cases of conscientious objection by the whole team, the coordinators referred the problem to the hospital administration or to another authorized service previously arranged, or explained to the patient that there were no physicians who perform a legal abortion in the institution, leaving patients with no referral and no solution to their problem. The research confirms the literature on this subject, and this situation may hamper the demand of women who want to interrupt their pregnancy. 16 Regardless of the condition that leads a professional to become a conscientious objector, they must be respected in this decision, as it is a medical right to refuse to perform medical acts, which although allowed by law, are contrary to their conscience. 17

The lack of structure and specific services identified in the research ( Table 1 ) besides conscientious objection by the whole team ( Fig. 2 ) and the non-performance of legal abortion ( Fig. 3 ) go against the Law 12,845/2013, 18 which defines mandatory and comprehensive assistance for victims of sexual violence. This law provides, in article 1, that the Unified Health System facilities must absolutely and promptly assist victims of sexual violence, with a multidisciplinary team providing various services, including in the event of legal abortion. This type of program represents an advance in humanized care and in the rights of women victims of sexual violence, 6 but the research statically revealed that the practice is still far from what is recommended by law.

As already studied in the text, conscientious objection is the medical right to oppose to perform procedures or services against moral, ethical, social or religious convictions. 19 In this perspective, the research identifies religion ( Table 2 ) as the most relevant reason why physicians are conscientious objectors. Among the several reasons that lead physicians to be conscientious objectors to carry out legal abortion, the literature cites social stigma 20 as the main obstacle to perform this procedure, even where the abortion is protected by law with policies, resolutions and protocols for it. These limitations encounter the ambiguities and insecurities of health workers to obey the law, either because they feel largely responsible for applying the eligibility criteria to avoid possible legal consequences; or for fear of being pejoratively labeled as abortionists and carrying the stigma of “agents of death.” There are also those who deny abortion care because they fear violence by politicians, the population and religious groups who are against abortion, including American and Brazilian obstetricians.

Brazil must increase relevance for conscientious objection, so that the population and government agencies become aware of the health care structures that support specific cases, such as legal abortion, thus providing real comprehensive assistance. Even if physicians are conscientious objectors to legal abortion, they must provide information about treatments they consider objectionable and must refer patients to professionals who are not objectors. 21

It should be noted that one of the scopes of the Ministry of Health is precisely to promote proper and humanized care for women in need of abortion, and among the basic principles for this goal are equality, freedom and human dignity, with granted access to comprehensive health care by the victim. 22 For health care professionals, humanized care implies abstracting moral, cultural, and religious convictions from their conduct, as well as other aspects that may influence on patient care; that is, their attitude must be guided by impartiality (justice) above all. This duality must be thoroughly discussed since it highlights and delineates the principle of justice. On one side of it, there is a woman who needs ethical care at its best, whose problem needs to be solved within the law, and, on the other side of it, there is a physician who refuses to perform this procedure for personal reasons, since they do not share the same convictions. In this case, bioethics must cover this controversial point, leading to conciliation and building bridges to rectify this situation, in which both rights must be protected. 23

Autonomy is the right to have decisions respected. Therefore, its lack can generate several conflicts, mainly in the biomedicine. To have complete autonomy, the individual must be in perfect condition to choose. When this is not possible, protective actions may be necessary and health professionals must guarantee it. 24 Since the research showed that 60.60% of the services interviewed in Minas Gerais State do not perform legal abortion, because their teams are all conscientious objectors to this medical act, it is of utmost importance to have a serious and resolute bioethical discussion to promote this issue in this state. Autonomy must be granted to women who opt for legal abortion and to physicians who refuse to perform such procedure.

The survey revealed that 85.70% of the interviewed services ( Fig. 1 ) have already dealt with women wishing to have a legal abortion. Due to these reasons, political actions aimed at promoting discussions and actions on the difficult and delicate problem of legal abortion are in need. Debates and arenas for bioethical reflection should favor changes in how health care institutions provide patient care so that, when dealing with women who wish and need to have a legal abortion, the professionals may experience less discomfort and feel less embarrassment in performing abortion, or have their right respected when they do not do so on moral grounds.

Even though there is an overlap between the physician and the moral subject, it is known that, before being health professionals who work for the State, they are part of a community that has its moral, cultural and religious precepts. For this reason, their whole life and history are based on duties of conscience, being able to choose what is right or wrong for their medical work. In addition to having the right not to perform procedures deemed wrong, the conscientious objector may also not want to inform the patients' rights nor refer them to another professional. From this point of view, the woman who has the right to abort would be totally unassisted and obstructed by moral justification. 24 25 The detailed identification of authorized services in the resolution of legal abortion and their intimate connection must be established for a real referral and counter-referral system so that patients can be optimally assisted, both from the point of view of humanization and of excellence in their care, providing a real comprehensive care for these women.

Abortion, in bioethics, is considered a persistent situation, seeming to be a very discussed and consolidated subject, but what actually happens in reality is an immense neglect in the care of women victims of sexual violence; it is for these reasons that bioethics cannot remain stagnant within reflections, convictions and theoretical and rhetorical discourses, while women are being violated not only by the situation of legal abortion, but also by the lack of proper assistance by multidisciplinary and interdisciplinary teams. 8

It is certain that bioethics is and always will be an arena for dialogue for all professionals confronted by this problem, so that they can intelligently and fully discuss it, promoting better patient-centered care, providing respect and comprehensive care. And it is expected that the physician can practice the medical profession with respect and rights safeguarded if there is conscientious objection to carry out abortion.

## Conclusion

According to the proposed objective, it is concluded that there is conscientious objection and it can significantly interfere in comprehensive health care for the victim of sexual violence and in the wish to terminate pregnancy in the state of Minas Gerais. After this research, it can be stated that it is common having patients who wish to terminate pregnancy, and that conscientious objection is expressive among the whole health care team, mainly due to ethical-religious influence. Discussions about health care, improvement of new structural models and updated accreditation by SES/MG are necessary due to the lack of structure and deficiency of services in the state of Minas Gerais, and conscientious objection to legal abortion. It is evident that the problem is not the criminalization, but the real lack of assistance, besides the lack of training to assist women entitled to legal abortion in Brazil. A lot of discussions on the legalization of abortion are important and must take place, but the government must reflect and invest to open care centers for victims of sexual violence, with a multidisciplinary team, adequate and personal service, having discretion and privacy, thus offering true humanized care, ceasing to be theoretical and rhetorical to be effective and comprehensive care.33 These specific centers are necessary for the problem of sexual violence and legal abortion in Brazil, thus leaving the drawing board and truly putting into practice in health care. The result of this study is expected to promote dialogues and debates not only in Minas Gerais State but also throughout Brazil, in addition to universities and their undergraduate and postgraduate courses, to encourage greater reflections on abortion. The patient shall be entirely welcomed and respected by medical professionals, being also respected conscientious objection, if it is present, and the government shall develop a referral and counter-referral system to overcome such a grave problem.
